# Longitudinal changes in the position and thickness of the peak peripapillary retinal nerve fiber layer in school children

**DOI:** 10.1007/s00417-025-06810-z

**Published:** 2025-03-25

**Authors:** Takehiro Yamashita, Hiroto Terasaki, Ryo Asaoka, Naoya Yoshihara, Naoko Kakiuchi, Taiji Sakamoto

**Affiliations:** 1 Department of Ophthalmology, Kagoshima University Graduate School of Medical and Dental Sciences, Kagoshima, Japan; 2 Department of Ophthalmology, Seirei Hamamatsu General Hospital, Shizuoka, Japan; 3 Seirei Christopher University, Shizuoka, Japan; 4 Nanovision Research Division, Research Institute of Electronics, Shizuoka University, Shizuoka, Japan; 5 The Graduate School for the Creation of New Photonics Industries, Shizuoka, Japan

**Keywords:** Peripapillary retinal nerve fiber layer thickness, Glaucoma, Longitudinal study, School myopia

## Abstract

**Purpose:**

This study investigated the relationship between changes in the position and thickness of the peak circumpapillary retinal nerve fiber layer (cpRNFL) and axial elongation in schoolchildren.

**Methods:**

This prospective cohort study involved the right eyes of 75 elementary school students examined over a period of six years (from the age of 8–9 years to 14–15 years). During the first and final years, all participants underwent optical axial length measurements, color fundus photography, and cpRNFL thickness measurements using optical coherence tomography. The supratemporal (ST) and infratemporal (IT) peak angles (ST and IT angle) were defined as those formed by the ST/IT peak position of the cpRNFL curve, the center of the optic disc, and the fovea. The RNFL thickness at the peaks (ST and IT thicknesses) was also determined. The Wilcoxon signed-rank test was used to compare the cpRNFL parameters and axial lengths in the first and final years.

**Results:**

The mean axial length was significantly longer in the final year (24.82 mm) than in the first year (23.34 mm). The mean ST and IT angles were significantly lower in the final year (67.6° and 58.2°) than in the first year (74.2° and 64.0°). The mean IT thickness was significantly greater in the final year (195.1 μm) than in the first year (185.0 μm); however, no significant changes in ST thickness were observed.

**Conclusion:**

The ST and IT peaks shifted toward the line connecting the fovea and the center of the optic disc between ages 8–9 and 14–15 years, and IT thickness increased. These changes indicate that nerve fibers are concentrated on the temporal side of the optic disc, especially in the IT area.

**Key Messages:**

*****What is known***:**

- The circumpapillary retinal nerve fiber layer (cpRNFL) in normal eyes exhibits a double-hump pattern, with individual variability in the position of the peaks. Additionally, the mechanisms underlying these differences remain unclear.

*****What is new***:**

- Eyes with greater axial elongation tended to have narrower supratemporal (ST) and infratemporal (IT) angles and increased IT thickness.
- Greater axial elongation during childhood growth caused a significant shift of the cpRNFL peaks toward the fovea and increased IT thickness.
- Based on the plate hypothesis, the shift and compression of nerve fibers during growth may serve as a potential predictor of normal-tension glaucoma onset in the future.

## Introduction

Glaucoma is an optic neuropathy characterized by irreversible loss of nerve fibers in the retinal ganglion cells [1]. Optical coherence tomography (OCT) can quantify nerve fiber loss, and this markedly improves the diagnosis and progression of glaucoma [2]. Circumpapillary retinal nerve fiber layer (cpRNFL) assessment can capture all retinal nerve fibers in a single scan image and allow visualization of the localized nerve fiber atrophy characteristic of glaucoma. Therefore, it has been used since the early development of OCT [3]. The cpRNFL in normal eyes is thickest on the supra- and infratemporal sides, forming what is known as the double-hump pattern [4]. In several cases of glaucoma, damage first occurs in these thick retinal nerve fiber bundles [5].

In contrast, the retinal nerve fiber trajectory varies from person to person; no two individuals have the same retinal nerve fiber trajectory [6]. For example, the peak angles of the supratemporal (ST) and infratemporal (IT) cpRNFLs (ST and IT angles) can be quantified using the angle relative to the line passing through the fovea and the center of the optic disc. Normal eyes with longer axial lengths tend to have narrower ST and IT angles [7]. Consequently, in normal-tension glaucoma, nerve fiber bundle defects tend to appear closer to the fovea in eyes with narrower ST and IT angles [8]. Additionally, false positives have been reported for normal eyes with ST and IT angles shifted toward the fovea when assessed using an OCT normative database [4, 9].

Thus, individual differences in the cpRNFL affect the diagnosis of glaucoma; however, how these individual differences arise remains unknown. Axial length measures approximately 16 mm at birth and gradually increases during the growth period. In emmetropic adults, it reaches approximately 24 mm [10]. This change represents a 1.5-fold increase in length but more than a three-fold increase in volume. During school age, the posterior eye is mainly elongated in the equatorial region, and a proportion of eyes change in shape from spherical to oval, resulting in progressive myopia [11, 12]. These changes in eye shape are presumed to be the main cause of individual differences in retinal nerve fiber trajectory. The cpRNFL thickness (cpRNFLT) in schoolchildren has been reported in cross-sectional studies [13, 14]; however, reports of long-term observational studies are limited [15]. Cross-sectional studies encompass generational and individual differences, as well as changes with age. In contrast, long-term observational studies can directly investigate age-related changes. Therefore, the purpose of this study was to clarify the changes in the angle and thickness of the ST and IT peaks of the cpRNFLT profile and their correlation with ocular axial elongation in school children using a long-term observational study design.

## Methods

### Ethics statement

The methodologies employed adhered to the principles outlined in the Declaration of Helsinki, and the study was approved by the Ethics Committee of Kagoshima University Hospital (Approval No. 170116(643)). Written informed assent and consent were obtained from all participants and their guardians. This study was registered in the University Hospital Medical Network Clinical Trials Registry (Registration No. UMIN000015239).

### Participants

This study was a longitudinal, prospective, observational investigation involving third-grade students aged 8 to 9 years during the initial examination [16]. The students were enrolled in the Elementary School of the Faculty of Education, Kagoshima University. Of the 144 eligible third-grade students, written informed consent was obtained from 122 students (87.4%) and their parents. The examinations took place during their first year from November 17 to December 18, 2014 (at the age of 8–9 years) and their final year in 2020 (at the age of 14–15 years). Only the right eye was assessed to avoid false confidence intervals and low P-values. The exclusion criteria included abnormal findings on fundus photography, systemic illnesses that affect the eyes, abnormal developmental history, previous trauma or ocular surgery, strabismus, noncooperation of the child, truancy, transfer to or enrollment in another junior high school, instances where the peripheral fundus area lacked clarity, and eyes with unmeasurable fundus and ocular parameters.

### Measurements of the angle and thickness of ST and IT peaks

**Fig. 1:**
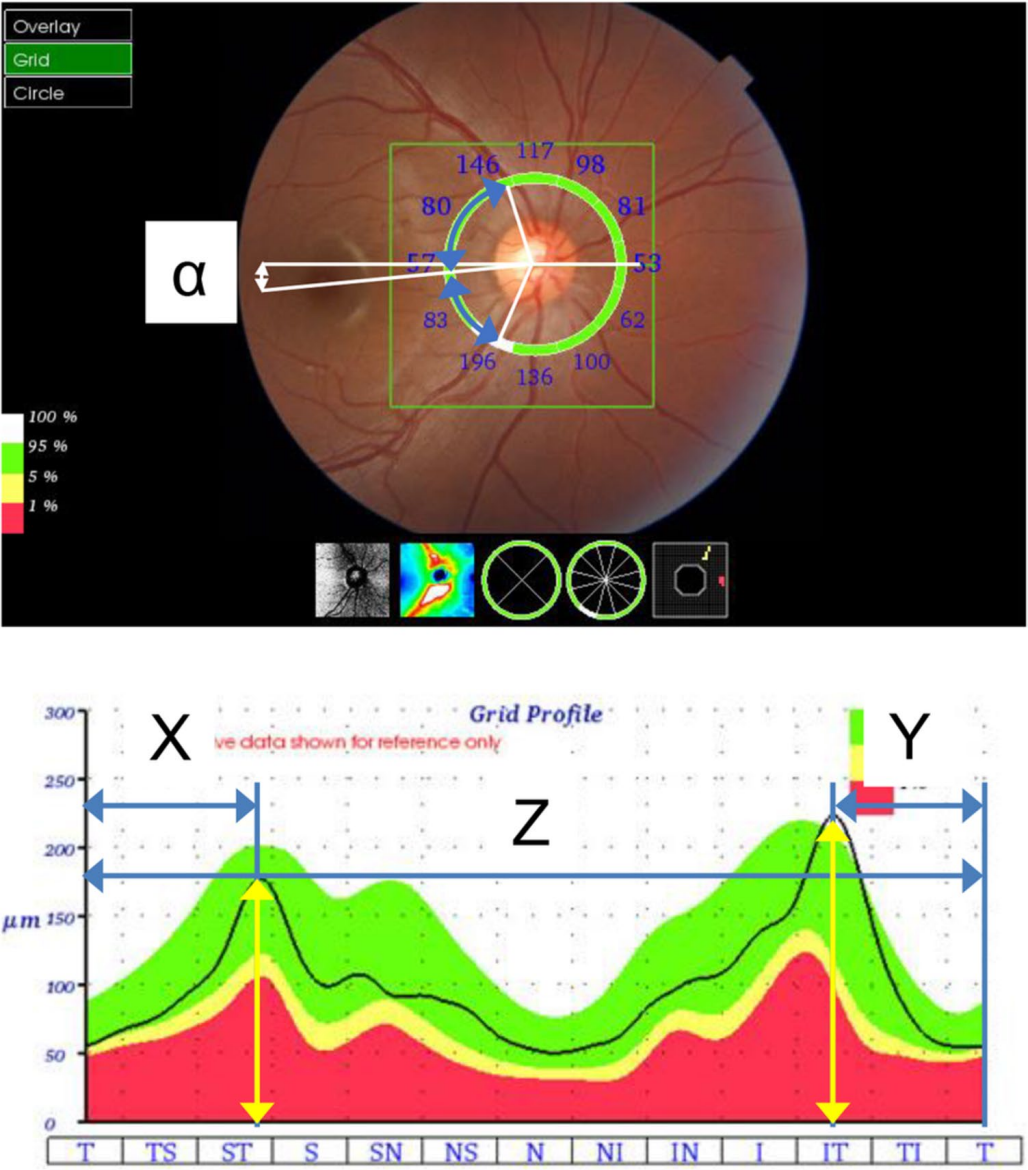
Measurements of the angle and thickness of the supratemporal (ST) and infratemporal (IT) peaks in the circumpapillary retinal nerve fiber layer (cpRNFL) curve. The length from left end to the ST cpRNFL peak (X) and right end to the IT cpRNFL peak (Y) were converted to an angular value by dividing it by the entire length (Z) and multiplying it by 360°. The angles of the ST and IT peaks (ST and IT angles) of the cpRNFL curve relative to the line connecting the fovea was calculated using the optic disc to fovea angle (α). The cpRNFL thickness of the peaks (ST and IT thicknesses) was determined using the cpRNFL thickness profile (yellow double arrows)

The axial length and corneal radius were measured using an OA-2000 Optical Biometer (Tomey, Nagoya, Japan). Color fundus photographs and cpRNFLT images were captured using 3D OCT-1 Maestro (Topcon, Tokyo, Japan). The scan type of the OCT was an Optic Disc Cube (6 mm × 6 mm) scan. Since the image size decreases with increasing axial length, a built-in magnification correction function in the OCT system was applied to adjust for this effect [17]. The repeatability and reproducibility of RNFL thickness measurements by the 3D OCT-1 Maestro Optic Disc Cube (6 mm × 6 mm) scans were excellent [18]. Therefore, the OCT scan was performed in a single measurement. To minimize variability due to misplacement of the measurement location and/or segmentation error, an experienced researcher (T.Y.) checked the locations of the disc barycenter and layer segmentation in all images using a previously described method [15]. The cpRNFLT profile was measured along a 3.4-mm-diameter circle centered on the disc center. Temporal, superior, nasal, inferior, and temporal (TSNIT) thickness curves were used to measure the ST and IT angles. We determined the peaks in the TSNIT thickness profile of the RNFL thickness analyses (Fig. 1). The length from the left end to the ST RNFL peak (Fig. 1X) and the right end to the IT RNFL peak (Fig. 1Y) were converted to an angular value by dividing it by the entire length (Fig. 1Z) and multiplying it by 360° [3]. The angles of the ST and IT peaks of the cpRNFL curve relative to the line connecting the fovea (ST and IT angles) were calculated using the optic disc to fovea angle (Fig. 1**α**) [7]. The RNFL thicknesses of the peaks (ST and IT thicknesses) were determined using the cpRNFLT profile.

### Statistical analysis

All statistical analyses were performed using SPSS Statistics 21 for Windows (IBM Corp., Armonk, New York, USA). The longitudinal changes in the axial length, ST and IT angles, and ST and IT thicknesses between the same students aged 8–9 years and 14–15 years were analyzed using the Wilcoxon signed-rank test. Correlations between the ST and IT angles, ST and IT thicknesses, and axial length at 8–9 or 14–15 years were analyzed using Spearman’s correlation. Correlations between the magnitudes of changes in the ST and IT angles, ST and IT thicknesses, and axial elongation were also analyzed using Spearman’s correlation. Statistical significance was set at P < 0.05.

## Results

**Fig. 2:**
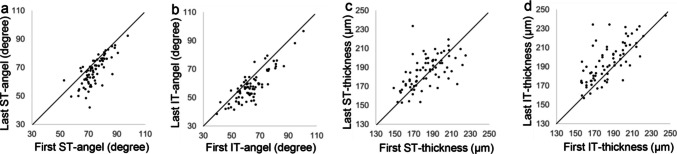
Scatterplot diagrams of the of the ST and IT angles (a, b) and thicknesses (c, d) in the first (at the age of 8–9 years) and final (at the age of 14–15 years) years. ST, supratemporal; IT, infratemporal

Of the 122 students initially enrolled, 14 were excluded due to difficulties in assessing the fundus and cpRNFL parameters, and 33 were excluded due to truancy, transfer, or enrollment in another junior high school. Consequently, data from 75 students (37 boys and 38 girls) were analyzed, covering the period from ages 8–9 to 14–15 years. The ocular parameters of the participants are shown in Table 1. Over the six-year period, the following significant changes were observed: their axial lengths significantly increased (*P* < 0.001), their ST and IT angles significantly narrowed (*P* < 0.001), and their IT thickness significantly increased (*P* < 0.001). The ST thicknesses of the students aged 8–9 and 14–15 years were not significantly different (*P* = 0.163). Scatterplot diagrams of the ST and IT angles (**a, b**) and thicknesses (**c, d**) in the first and final years are shown in Fig. 2.

**Table 1:** Demographic information of the right eyes of the 75 eligible subjects

	8.5 years old	14.5 years old	Change	*P* value
Axial length (mm)	23.34 ± 0.92	24.82 ± 1.14	1.48 ± 0.50	< 0.001
ST angle (degree)	74.2 ± 7.7	67.6 ± 9.8	−6.5 ± 6.4	< 0.001
IT angle (degree)	64.0 ± 11.1	58.2 ± 11.0	−5.9 ± 6.0	< 0.001
ST thickness (μm)	183.6 ± 18.1	186.1 ± 17.7	2.5 ± 17.1	0.16
IT thickness (μm)	185.0 ± 21.5	195.1 ± 20.5	10.0 ± 16.2	< 0.001

*ST* supratemporal, *IT* infratemporal

**Fig. 3:**
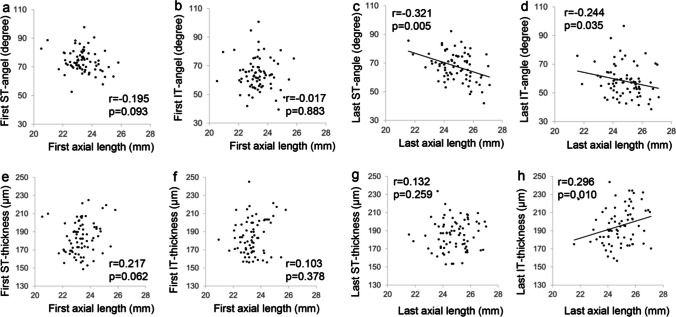
Relationship between ST and IT angles and axial length in the first (at the age of 8–9 years) and final (at the age of 14–15 years) years. (a, b, c, d) Relationship between ST and IT thicknesses and axial length in the first (at the age of 8–9 years) and final (at the age of 14–15 years) years. (e, f, g, h) ST, supratemporal; IT, infratemporal

The relationships between the ST and IT angles and axial lengths in the first and final years are shown in Fig. 3. In the first year, the ST (r = −0.195, *p* = 0.093) (**a**) and IT (r = −0.017, *p* = 0.883) (**b**) angles were not significantly associated with axial length. In the final year, the ST (r = −0.321, *p* = 0.005) (**c**) and IT (r = −0.244, *p* = 0.035) (**d**) angles were significantly and negatively associated with axial length. The relationships between ST and IT thicknesses and axial lengths in the first and final years are shown in Fig. 3. In the first year, the ST (r = 0.217, *p* = 0.062) (**e**) and IT (r = 0.103, *p* = 0.378) (**f**) thicknesses were not significantly associated with axial length. In the final year, there was no significant relationship between ST thickness and axial length (r = 0.132, *p* = 0.259) (**g**); however, IT thickness was significantly and positively associated with axial length (r = 0.296, *p* = 0.010) (**h**).

**Fig. 4:**
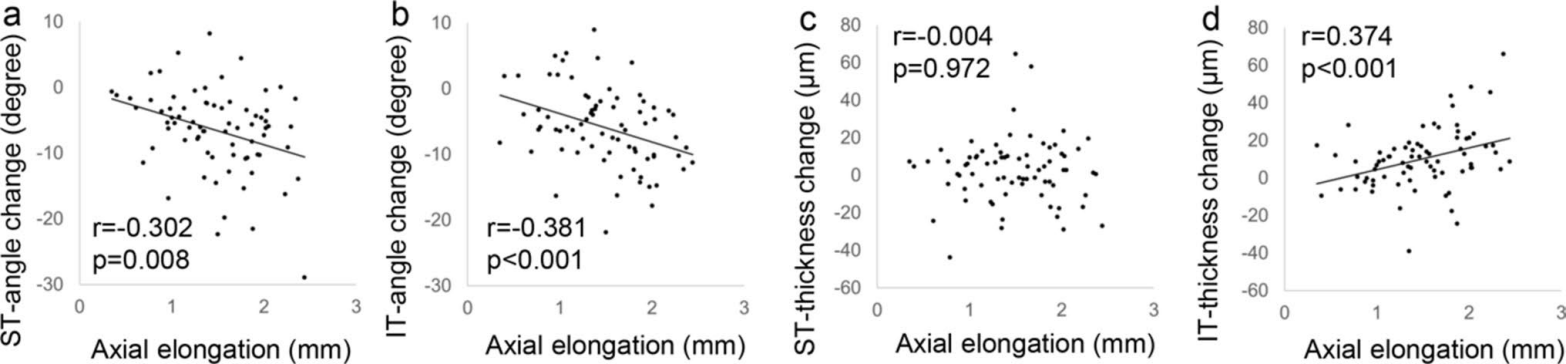
Relationship between the magnitudes of changes in the ST and IT angles (a, b) and thicknesses (c, d) and axial elongation over 6 years. ST, supratemporal; IT, infratemporal

The relationships between the changes in the ST and IT peaks and ST and IT thicknesses and axial elongation over the six-year period are shown in Fig. 4. Changes in the ST (r = −0.302, p = 0.008) (**a**) and IT (r = −0.381, *p* < 0.001) (**b**) angles were significantly and negatively associated with axial elongation. There was no significant relationship between the changes in the ST thickness and axial elongation (r = −0.004, *p* = 0.972) (**c**); however, changes in the IT thickness were significantly and positively associated with axial elongation (r = 0.374, *p* < 0.001) (**d**).

**Fig. 5:**
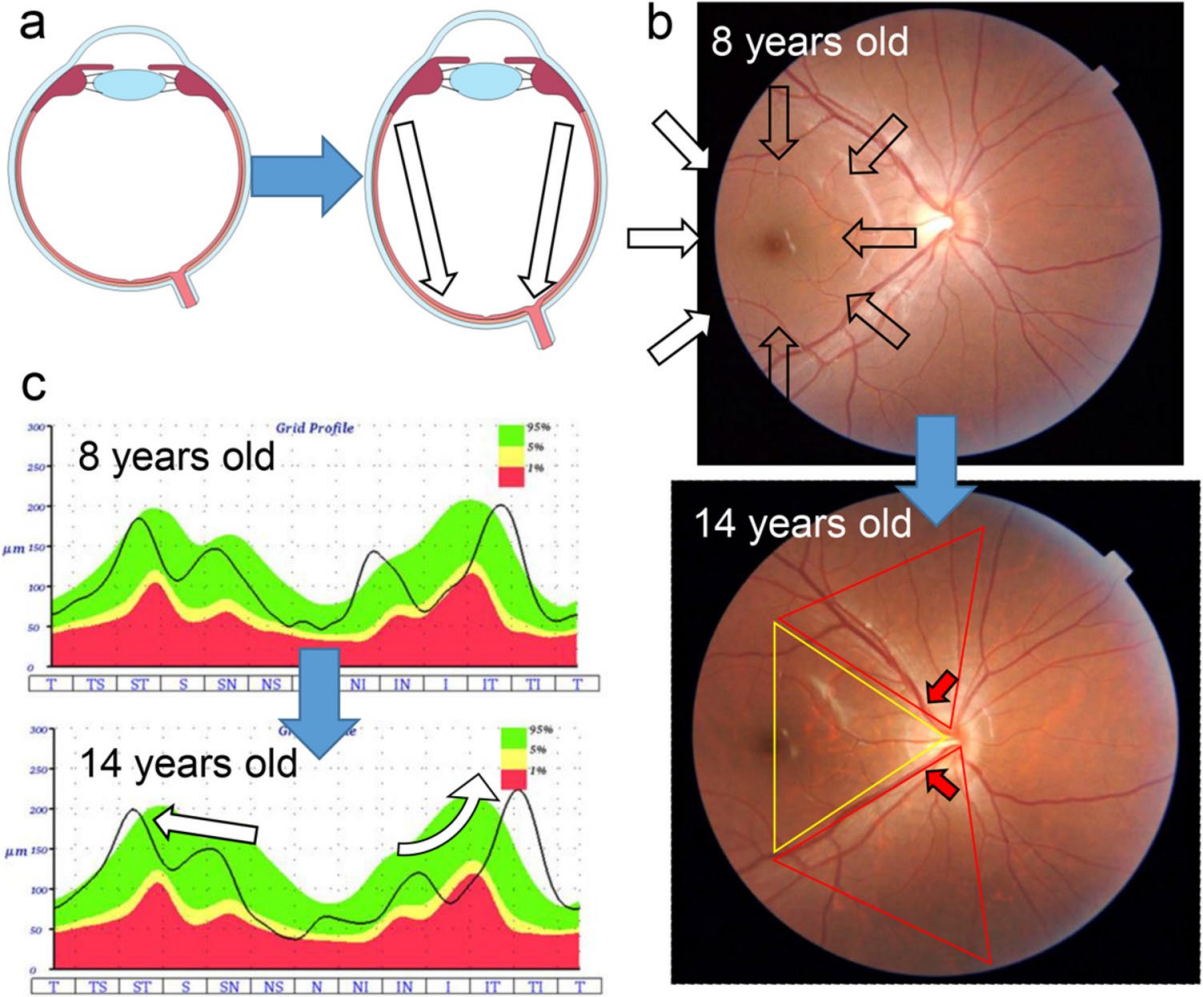
Fundus and cpRNFL changes of representative case with oval eye elongation. (a) Oval axial elongation pattern of the eye wall in schoolchildren. (b) White arrows show the direction of change along with oval eye elongation. (c) A representative case with ST- and IT-peak shifts toward the fovea and increasing IT thickness. These shifts cause compression of the temporal side of the optic disc (triangles and red arrows). The fundus image size of the 8-year-old was reduced using Bennett’s formula. cpRNFL, circumpapillary retinal nerve fiber layer; IT, infratemporal; ST, supratemporal

In a representative case, the ST and IT peaks shifted toward the fovea, and the IT thickness increased (Fig. 5). At 8 and 14 years of age, the students had axial lengths of 24.57 μm and 26.44 mm, ST angles of 67.8° and 54.8°, IT angles of 45.7° and 42.1°, ST thicknesses of 185.2 μm and 200.0 μm, and IT thicknesses of 203.4 μm and 224.9 μm, respectively. White arrows show the direction of change along with oval eye elongation. The fundus image size of the 8-year-old was reduced using Bennett’s formula [19].

## Discussion

The results showed that the axial length increased significantly from the age of 8–9 years to 14–15 years, the ST and IT angles significantly narrowed, and the IT thickness significantly increased. These results suggest that the peaks and thicknesses of the cpRNFL change with eye growth. During infancy, both the anterior and posterior segments of the eye expand spherically. Therefore, even if the axial length increases, this is offset by the increase in the corneal curvature radius, and the impact of refractive myopia may not be significant [11]. During school age, the posterior segment elongates (especially along the equator), and the eyeball becomes egg-shaped from front to back. The focal point of light from infinity shifts forward from the retina, causing myopia progression [12]. Figure 5 shows the portion of the eye elongated in the direction of the white arrow in the fundus photograph during this egg-shaped enlargement. The optic disc becomes tilted [20], nerve fibers are elevated on the nasal side of the optic disc [21], parapapillary atrophy (PPA) occurs on the temporal side [22], and the arcade vessels [16] and retinal nerve fibers shift toward the fovea [4]. We also observed that the thickness of the IT cpRNFL peak increased during this period.

These fundus changes are geologically similar to the changes in the tectonic plates of the Earth. Folding is a phenomenon characterized by the pushing of the plates against each other, causing continents to rise; the ongoing state of folding is defined as an active fold [23]. In the fundus, the ST and IT plates (red triangles in Fig. 5) press against the macular plate (yellow triangle in Fig. 5) during myopia progression, and the peaks of the cpRNFLT shift and rise like folds of tectonic plates. Nerve fibers get compressed on the temporal side of the optic disc (such as in active folds) if these changes are large and continue into adulthood, and the optic disc gets compressed in the direction of the red arrow, shown in Fig. 5. The optic disc, blood vessels, and nerves in the optic disc are expected to be compressed by increased or fluctuating intraocular pressure, causing local nerve fiber damage.

Normal-tension glaucoma causes local nerve fiber damage, especially on the temporal side of the optic disc, and myopia is a known risk factor. In Asia, including Japan, myopia is common, and normal-tension glaucoma is observed at a high rate [24]. In normal-tension glaucoma, optic nerve damage progresses locally. In a some eyes, progression does not occur even without treatment [25]. In these eyes, nerve fiber bundle defects occur on the temporal side of the optic disc; however, glaucoma does not progress on the nasal side. These phenomena may be explained by the “plate hypothesis” described above during oval enlargement with the progression of myopia. Long-term observation of the axial length of adult eyes with normal-tension glaucoma shows that it increases [26], and the temporal PPA expands [27]. These indicate that ocular enlargement progresses slowly in adult eyes. In addition, the expansion of the PPA has been associated with glaucoma progression [27], suggesting that glaucoma may progress in adult eyes with progressive oval enlargement. These studies suggest that optic nerve damage only progresses in areas with nerve fiber compression on the temporal side. As this egg-shaped enlargement of the eye causes myopia, local glaucomatous optic nerve damage occurring at the compressed part of the nerve fibers may be called "myopic glaucoma.” This myopic glaucoma hypothesis is a prediction in theoretical physics and cannot be directly proven by the data in this study.

Figure 2 shows that the ST and IT angles decrease with growth; however, the magnitudes differ. Conversely, the angles widen in some eyes. In addition, the ST thickness did not change significantly but increased in some eyes and decreased in others over six years.

In eyes with increasing ST and IT angles, the IT and ST thicknesses reduce, and spherical eyeball enlargement may continue even at school age. No two eyes are the same, and the shape of the eyeball differs from person to person. Considering these individual differences, it is not surprising that glaucomatous optic nerve damage occurs in various locations, such as the ST or temporal regions of the optic disc. Individual differences in eyeball shape indicate that there are individual differences in the deformation of the optic disc when intraocular pressure increases or fluctuates, which may be related to individual differences in the location of glaucomatous optic neuropathy. In glaucomatous eyes, the original peak cannot be determined due to a reduction in nerve fibers; however, the course of the peak matches the course of arcade blood vessels [7]. A study investigating the course of arcade blood vessels in normal-tension glaucoma reported that the proximity of the retinal artery to the fovea was associated with the proximity of the nerve fiber layer defect [8]. Thus, the plate hypothesis can explain various characteristics of normal-tension glaucoma; however, long-term observational studies from childhood to the onset of glaucoma in adults are needed to validate these findings.

In this study, axial length was not significantly correlated with ST or IT angle or thickness at the age of 8–9 years. In the amount of change over six years, the greater axial elongation was associated with larger ST and IT angles, greater proximity to the fovea, and greater IT thickness. As a result, eyes with greater axial lengths tended to have narrower ST and IT angles and greater IT thickness at the age of 14–15 years. These results indicate that the greater axial elongation was associated with greater shift and compression of the nerve fibers. The compression of nerve fibers as the cause of normal-tension glaucoma is consistent with reports that higher myopia was associated with a higher risk of normal-tension glaucoma. Nerve fiber shifts during childhood may be a promising predictor of normal-tension glaucoma onset [5, 21].

This study has some limitations. It focused on Japanese children, and different trends may be observed for other ethnic groups, especially those with lower incidence of myopia and normal-tension glaucoma. Another limitation was the small sample size. Investigations involving larger cohorts are required. Unlike continental plates, the eye lacks fixed and mobile boundaries. However, its surface deforms in a manner similar to tectonic activity, which inspired the “plate hypothesis” terminology to facilitate conceptualization. Proving this myopic glaucoma hypothesis will be the subject of future research, and we reiterate that the absence of such proof remains a limitation of this study.

In conclusion, this long-term observational study showed no significant correlations between axial length and the angles and thicknesses of the peak cpRNFL at 8–9 years of age. However, eyes with greater axial lengths tended to have narrower ST and IT angles and greater IT thickness at 14–15 years of age. Furthermore, greater axial elongation caused a larger shift of the cpRNFL peaks toward the fovea [4] and increased the IT RNFLT over six years. Individual differences in the distribution of these nerve fibers and changes during growth may cause local compression or deviation of the nerve fibers.

## Abbreviations

cpRNFL: Circumpapillary retinal nerve fiber layer
cpRNFLT: Circumpapillary retinal nerve fiber layer thickness
IT: Infratemporal
PPA: Parapapillary atrophy
RNFL: Retinal nerve fiber layer
ST: Supratemporal
TSNIT: Temporal, superior, nasal, inferior, and temporal

## References

[1] Quigley HA, Addicks EM, Green WR (1982) Optic nerve damage in human glaucoma. III. Quantitative correlation of nerve fiber loss and visual field defect in glaucoma, ischemic neuropathy, papilledema, and toxic neuropathy. Arch Ophthalmol 100:135–146. 10.1001/archopht.1982.01030030137016 7055464 10.1001/archopht.1982.01030030137016

[2] Hood DC, La Bruna S, Tsamis E, Thakoor KA, Rai A, Leshno A, de Moraes CGV, Cioffi GA, Liebmann JM (2022) Detecting glaucoma with only OCT: Implications for the clinic, research, screening, and AI development. Prog Retin Eye Res 90:101052. 10.1016/j.preteyeres.2022.101052 35216894 10.1016/j.preteyeres.2022.101052

[3] Blumenthal EZ, Williams JM, Weinreb RN, Girkin CA, Berry CC, Zangwill LM (2000) Reproducibility of nerve fiber layer thickness measurements by use of optical coherence tomography. Ophthalmology 107:2278–2282. 10.1016/s0161-6420(00)00341-9 11097610 10.1016/s0161-6420(00)00341-9

[4] Shin JW, Uhm KB, Seong M, Lee DE (2014) Retinal nerve fiber layer volume measurements in healthy subjects using spectral domain optical coherence tomography. J Glaucoma 23:567–573. 10.1097/IJG.0b013e3182948673 23970339 10.1097/IJG.0b013e3182948673

[5] Garway-Heath DF, Poinoosawmy D, Fitzke FW, Hitchings RA (2000) Mapping the visual field to the optic disc in normal tension glaucoma eyes. Ophthalmology 107:1809–1815. 10.1016/s0161-6420(00)00284-0 11013178 10.1016/s0161-6420(00)00284-0

[6] Lamparter J, Russell RA, Zhu H, Asaoka R, Yamashita T, Ho T, Garway-Heath DF (2013) The influence of intersubject variability in ocular anatomical variables on the mapping of retinal locations to the retinal nerve fiber layer and optic nerve head. Invest Ophthalmol Vis Sci 54:6074–6082. 10.1167/iovs.13-11902 23882689 10.1167/iovs.13-11902

[7] Yamashita T, Asaoka R, Tanaka M, Kii Y, Yamashita T, Nakao K, Sakamoto T (2013) Relationship between position of peak retinal nerve fiber layer thickness and retinal arteries on sectoral retinal nerve fiber layer thickness. Invest Ophthalmol Vis Sci 54:5481–5488. 10.1167/iovs.12-11008 23847316 10.1167/iovs.12-11008

[8] Yamashita T, Nitta K, Sonoda S, Sugiyama K, Sakamoto T (2015) Relationship between location of retinal nerve fiber layer defect and curvature of retinal artery trajectory in eyes with normal tension glaucoma. Invest Ophthalmol Vis Sci 56:6190–6195. 10.1167/iovs.15-17119 26416093 10.1167/iovs.15-17119

[9] Yamashita T, Kii Y, Tanaka M, Yoshinaga W, Yamashita T, Nakao K, Sakamoto T (2014) Relationship between supernormal sectors of retinal nerve fibre layer and axial length in normal eyes. Acta Ophthalmol 92:e481–e487. 10.1111/aos.12382 24655430 10.1111/aos.12382

[10] Lin H, Lin D, Chen J, Luo L, Lin Z, Wu X, Long E, Zhang L, Chen H, Chen W, Zhang B, Liu J, Li X, Chen W, Liu Y (2016) Distribution of axial length before cataract surgery in Chinese pediatric patients. Sci Rep 6:23862. 10.1038/srep23862 27022004 10.1038/srep23862 PMC4810521

[11] Mutti DO, Hayes JR, Mitchell GL, Jones LA, Moeschberger ML, Cotter SA et al (2007) Refractive error, axial length, and relative peripheral refractive error before and after the onset of myopia. Invest Ophthalmol Vis Sci 48(6):2510–2519. 10.1167/iovs.06-0562 17525178 10.1167/iovs.06-0562 PMC2657719

[12] Yip VC, Pan CW, Lin XY, Lee YS, Gazzard G, Wong TY, Saw SM (2012) The relationship between growth spurts and myopia in Singapore children. Invest Ophthalmol Vis Sci 53:7961–7966. 10.1167/iovs.12-10402 23150611 10.1167/iovs.12-10402

[13] Yao Y, Fu J, Li L, Chen W, Meng Z, Su H, Dai W (2021) Retinal and circumpapillary nerve fiber layer thickness and associated factors in children. Eye (Lond) 35:2802–2811. 10.1038/s41433-020-01313-z 33239762 10.1038/s41433-020-01313-z PMC8452704

[14] Rajjoub RD, Trimboli-Heidler C, Packer RJ, Avery RA (2015) Reproducibility of retinal nerve fiber layer thickness measures using eye tracking in children with nonglaucomatous optic neuropathy. Am J Ophthalmol 159:71-77.e1. 10.1016/j.ajo.2014.09.029 25256068 10.1016/j.ajo.2014.09.029 PMC4455020

[15] Ha A, Kim YK, Baek SU, Kim JS, Jeoung JW, Park KH (2022) Longitudinal changes of circumpapillary retinal nerve fiber layer thickness profile during childhood myopia progression. Sci Rep 12:2555. 10.1038/s41598-022-06489-w 35169209 10.1038/s41598-022-06489-w PMC8847345

[16] Yamashita T, Terasaki H, Yoshihara N, Kii Y, Uchino E, Sakamoto T (2018) Relationship between retinal artery trajectory and axial length in Japanese school students. Jpn J Ophthalmol 62:315–320. 10.1007/s10384-018-0572-y 29442204 10.1007/s10384-018-0572-y

[17] Hirasawa K, Yamaguchi J, Nagano K, Kanno J, Kasahara M, Shoji N (2022) Structure-function relationships and glaucoma detection with magnification correction of OCT angiography. Ophthalmol Sci 2:100120. 10.1016/j.xops.2022.100120 36249704 10.1016/j.xops.2022.100120 PMC9562297

[18] Hou H, El-Nimri NW, Durbin MK, Arias JD, Moghimi S, Weinreb RN (2023) Agreement and precision of wide and cube scan measurements between swept-source and spectral-domain OCT in normal and glaucoma eyes. Sci Rep 13:15876. 10.1038/s41598-023-43230-7 37741895 10.1038/s41598-023-43230-7 PMC10517954

[19] Bennett AG, Rudnicka AR, Edgar DF (1994) Improvements on Littmann’s method of determining the size of retinal features by fundus photography. Graefes Arch Clin Exp Ophthalmol 232:361–367. 10.1007/BF00175988 8082844 10.1007/BF00175988

[20] Sameshima S, Yamashita T, Terasaki H, Asaoka R, Yoshihara N, Kakiuchi N, Sakamoto T (2024) Longitudinal changes of funduscopic optic disc size, color and cup-to-disc ratio in school children. Int J Retina Vitreous 10:51. 10.1186/s40942-024-00570-4 39054561 10.1186/s40942-024-00570-4 PMC11270882

[21] Fujiwara K, Yamashita T, Terasaki H, Nakao K, Sakamoto T (2024) Quantification of peripapillary nerve fibre elevation and its association with axial length, optic disc tilt, and parapapillary atrophy area in young, healthy eyes. Eye (Lond) 38:1112–1117. 10.1038/s41433-023-02827-y 37968515 10.1038/s41433-023-02827-y PMC11009348

[22] Jonas JB, Jonas RA, Bikbov MM, Wang YX, Panda-Jonas S (2023) Myopia: Histology, clinical features, and potential implications for the etiology of axial elongation. Prog Retin Eye Res 96:101156. 10.1016/j.preteyeres.2022.101156 36585290 10.1016/j.preteyeres.2022.101156

[23] Fleury MJ (1964) The description of folds. Proc Geol Assoc 75(4):461–492. 10.1016/S0016-7878(64)80023-7

[24] Kawase K, Tomidokoro A, Araie M, Iwase A, Yamamoto T, Tajimi Study Group, Japan Glaucoma Society (2008) Ocular and systemic factors related to intraocular pressure in Japanese adults: The Tajimi study. Br J Ophthalmol 92:1175–1179. 10.1136/bjo.2007.128819 18669541 10.1136/bjo.2007.128819

[25] Anderson DR, Drance SM, Schulzer M, Collaborative Normal-Tension Glaucoma Study Group (2001) Natural history of normal-tension glaucoma. Ophthalmology 108:247–253. 10.1016/s0161-6420(00)00518-2 11158794 10.1016/s0161-6420(00)00518-2

[26] Yanagisawa M, Yamashita T, Matsuura M, Fujino Y, Murata H, Asaoka R (2018) Changes in axial length and progression of visual field damage in glaucoma. Invest Ophthalmol Vis Sci 59:407–417. 10.1167/iovs.17-22949 29351357 10.1167/iovs.17-22949

[27] Bak E, Ha A, Kim YW, Lee J, Han YS, Kim YK, Jeoung JW, Park KH (2020) Ten years and beyond longitudinal change of ß-zone parapapillary atrophy: Comparison of primary open-angle glaucoma with normal eyes. Ophthalmology 127:1054–1063. 10.1016/j.ophtha.2020.01.057 32222399 10.1016/j.ophtha.2020.01.057

